# Pancreatic Ductal Adenocarcinoma Contains an Effector and Regulatory Immune Cell Infiltrate that Is Altered by Multimodal Neoadjuvant Treatment

**DOI:** 10.1371/journal.pone.0096565

**Published:** 2014-05-02

**Authors:** Kendall C. Shibuya, Vikas K. Goel, Wei Xiong, Jonathan G. Sham, Seth M. Pollack, Allison M. Leahy, Samuel H. Whiting, Matthew M. Yeh, Cassian Yee, Stanley R. Riddell, Venu G. Pillarisetty

**Affiliations:** 1 Clinical Research Division, Fred Hutchinson Cancer Research Center, Seattle, Washington, United States of America; 2 Department of Medicine, University of Washington, Seattle, Washington, United States of America; 3 Department of Pathology, University of Washington, Seattle, Washington, United States of America; 4 Department of Surgery, University of Washington, Seattle, Washington, United States of America; 5 Surgical Oncology, Seattle Cancer Care Alliance, Seattle, Washington, United States of America; 6 Department of Pathology and Laboratory Medicine, University of Calgary, Calgary, Alberta, Canada; The Liverpool Cancer Research UK Centre, United Kingdom

## Abstract

**Objective:**

The immune response to pancreatic ductal adenocarcinoma (PDA) may play a role in defining its uniquely aggressive biology; therefore, we sought to clearly define the adaptive immune infiltrate in PDA.

**Design:**

We used immunohistochemistry and flow cytometry to characterize the immune infiltrate in human PDA and compared our findings to the patients’ peripheral blood.

**Results:**

In contrast to the myeloid cell predominant infiltrate seen in murine models, T cells comprised the majority of the hematopoietic cell component of the tumor stroma in human PDA. Most intratumoral CD8^+^ T cells exhibited an antigen-experienced effector memory cell phenotype and were capable of producing IFN-γ. CD4^+^ regulatory T cells (Treg) and IL-17 producing T helper cells were significantly more prevalent in tumor than in blood. Consistent with the association with reduced survival in previous studies, we observed higher frequencies of both myeloid cells and Treg in poorly differentiated tumors. The majority of intratumoral T cells expressed the co-inhibitory receptor programmed death-1 (PD-1), suggesting one potential mechanism through which PDA may evade antitumor immunity. Successful multimodal neoadjuvant therapy altered the immunoregulatory balance and was associated with reduced infiltration of both myeloid cells and Treg.

**Conclusion:**

Our data show that human PDA contains a complex mixture of inflammatory and regulatory immune cells, and that neoadjuvant therapy attenuates the infiltration of intratumoral cells associated with immunosuppression and worsened survival.

## Introduction

Pancreatic ductal adenocarcinoma (PDA) remains one of the most rapidly fatal human malignancies.[1] Major advances in immunotherapy of a variety of human cancers are in part derived from a more rigorous understanding of the intricate relationship between a progressing tumor and the host immune response. In several human malignancies, including PDA, T cell infiltration of the tumor correlates with an improved prognosis despite the inhibitory effects of regulatory T cells (Treg), myeloid cells, cytokines and tumor associated ligands that often cohabitate the tumor microenvironment.[2]–[4].

Our understanding of the immune environment in pancreatic cancer has been influenced and enhanced by the development of genetically engineered mouse models (GEMM).[5] Clark reported a leukocyte infiltrate that paralleled disease progression and was predominately comprised of immunosuppressive cells including tumor-associate macrophages (TAM), myeloid derived suppressor cells (MDSC) and regulatory T cells (Treg), but few effector cells.[6] More recent studies have found that intratumoral T cells in Kras-driven GEMM are rare in the absence of treatment, owing to high levels of MDSC recruited by tumor-derived GM-CSF.[7]–[9] These findings have led to the general conclusion that PDA does not trigger an adaptive immune response.

A potential limitation of GEMM of PDA for understanding interactions with host immunity is the rapidity with which tumors develop after oncogene activation compared to the lengthy genetic evolution of human PDA.[10] Human studies using immunohistochemical (IHC) staining of tumor tissue or flow cytometry of peripheral blood alone have reported some similarities to GEMM including frequent intratumoral Treg,[11]–[13] TAM,[14] and MDSC,[1], [15], [16] and elevated systemic levels of Treg.[2]–[4], [12], [17] In contrast, there is also some evidence for a role of adaptive immunity in human PDA, including the presence of inflammatory IL-17 producing T helper (Th17) cells,[5], [18], [19] a CD8^+^ T cell infiltrate that correlates with MHC class I expression on tumor cells,[6], [20] and detection of functional tumor-reactive T cells in blood and bone marrow of PDA patients.[7]–[9], [21] High levels of tumor infiltrating CD8^+^ and CD4^+^ T cells with a low proportion of Treg have also correlated significantly with improved survival in human PDA.[2], [10], [22] Thus, these studies of human tissue suggest great variability in the composition of the immune infiltrate in pancreatic cancer.

In this study, we precisely characterized the adaptive immune infiltrate in patients with PDA, a proportion of whom underwent multimodal neoadjuvant treatment with chemotherapy and chemoradiotherapy. We used IHC to identify and localize T cells and myeloid cells in PDA tumors, and performed flow cytometry on single cell suspensions of both tumor and peripheral blood on a subset of patients. Since immune checkpoint blockade has shown promise in the treatment of other malignancies,[11]–[13], [23], [24] we examined whether the inhibitory receptor programmed death-1 (PD-1) was upregulated on T cells locally or systemically, and found that, in contrast to T cells in the peripheral blood, the majority of T cells in the PDA tumor microenvironment express PD-1. We also demonstrated that multimodal neoadjuvant therapy reduces the infiltration of PDA tumors by immunosuppressive cell types associated with reduced survival.

## Materials and Methods

### Ethics Statement

All investigations performed in relation to this manuscript were conducted according to the principles expressed in the Declaration of Helsinki. Fresh tumor and blood were procured from patients undergoing pancreatic resection for PDA, who provided written informed consent under a research protocol approved by the Cancer Consortium Institutional Review Board (CC-IRB) at the Fred Hutchinson Cancer Research Center. Tissue blocks were gathered under a separate CC-IRB-approved protocol for which there was a waiver of consent, as the study was considered to be of minimal risk since the tissue and data were collected solely for non-research purposes.

### Neoadjuvant Therapy

Neoadjuvant chemotherapy (GTX), typically consisted of three cycles of gemcitabine 750 mg/m^2^ IV, docetaxel 30 mg/m^2^ IV and capecitabine 750 mg/m^2^ PO.[14], [25] Within 14 days of completing the third cycle of chemotherapy, chemoradiotherapy (300 centigray over 10 fractions, intensity modulated radiation therapy plus oxaliplatin 60 mg/m^2^ IV and capecitabine 650 mg/m^2^ PO) was administered to the majority of patients. Primary tumor resection was performed 2–6 weeks later, following restaging evaluation. Blood and tissue were collected at the time of resection.

### Preparation of Tissue and Blood Specimens

Tumors were collected directly from Pathology immediately after margin assessment and were placed into cold media (RPMI 1640 media with 10% fetal bovine serum(Gemini Bio-Products) and 1% Penicillin/Streptomycin(Gibco) and transported on ice. To make single-cell suspensions, we minced tumors and incubated at 37°C for 30–45 minutes in an enzymatic cocktail(DNAse(0.1 mg/mL, Roche), collagenase type IV (1 mg/mL) and hyaluronidase(0.1 mg/mL, Worthington) in RPMI 1640(Sigma). This was passed through a 70 µM filter, washed, counted and either used immediately for flow cytometry or cryopreserved in media+10% DMSO for later analysis.

Peripheral blood(10–12 mL) was collected in heparin-containing tubes from each patient at the time of surgery and transported on ice. Blood was mixed with an equal volume of 1 mM PBS/EDTA, layered over 15 mL of histopaque, and centrifuged at 2,000 rpm for 20 minutes to isolate the leukocyte population.

### Immunohistochemistry

Slides were de-paraffinized in xylene and rehydrated through graded ethanol followed by heat mediated antigen retrieval using Lab Vision HIER L(Fisher). IHC was performed in two or three sequential incubations with one primary antibody followed by host-matched secondary polymer reagent and color substrate. Primary antibodies used were: CD3(Dako), CD11b(Epitomics), CD4, MIP-1alpha and CD8(ThermoFisher) and FOXP3(eBioscience). Secondary reagents were ImmPress Rabbit HRP and Mouse HRP(Vector Laboratories). Color development was performed using Quanto DAB(Fisher) brown, Vector VIP purple and Vector SG gray-blue. Slides were counterstained with hematoxylin as appropriate, dehydrated and mounted.

### Evaluation of Histology

Tissue sections were analyzed by pathologists and scientists (MY, WX and VKG) blinded to the patients’ history and prior pathologic assessment. Images were acquired using a Nuance EX Multispectral Tissue Imaging Systems (Perkin Elmer, USA) and analyzed using inForm software (Perkin Elmer) to quantify CD8^+^, CD4^+^, CD3^+^, CD11b^+^ and FOXP3^+^ cells, and measure staining intensity of GM-CSF.

### Flow Cytometry

Tumor and blood were stained without stimulation for the following surface markers: CD3, PD-1, CD45RO, CD8, CD62L, CD28 (BD), and for CD19 and CD4(eBioscience). Cytokine production was stimulated with phorbol 12-myristate 12-acetate(PMA, 50 ng/mL) and ionomycin(1 mM) in the presence of a Golgi blocker(Brefeldin A, 1 mL/mL) for 4–12 hours for intracellular cytokine analysis. All samples were labeled with live/dead stain (Invitrogen) prior to antibody labeling. To detect IL-17 producing T cells and Treg, we labeled the cells before fixing and permeabilizing(BioLegend FOXP3 Fix/Perm Buffer set) with CD3, CD4, CD127(eBiosciences), CD8, and CD25(BD), and then staining for IL-17(eBioscience) and FOXP3(BioLegend).

IFN-γ production was analyzed using a separate panel by labeling the cells with CD45, CD3, CD16, CD19(BD), and CD56(BioLegend), before fixing and permeabilizing the cells with the eBioscience fix/perm buffer set and then staining with IFN-γ (eBioscience). Flow cytometry for all samples and controls was conducted using an LSRII flow cytometer(BD). Data analysis and compensation using single-color controls were performed using FlowJo(Tree Star, Ashland, OR).

### Statistical Analysis

Values in graphs represent mean ± SEM. Differences between groups were determined using parametric two-tailed t-tests (paired or unpaired, as appropriate). Correlation was determined using Pearson’s method. Survival was graphed based upon the Kaplan-Meier method and survival curves were compared using the log-rank test. P-values ≤0.05 were considered statistically significant. Graphs and significance calculations were made using Prism 6.0(GraphPad Software, La Jolla, CA).

## Results

### T cells Dominate the Immune Infiltrate in PDA Despite Consistent Expression of GM-CSF by Cancer Cells

An inflammatory infiltrate in PDA is frequently noted, however its composition and the relationship of specific subsets of infiltrating immune cells to tumor cells are incompletely defined. Studies in GEMM of PDA have demonstrated that tumor-derived GM-CSF promotes the development of a dense myeloid infiltrate that suppresses infiltration of T cells within the tumor microenvironment.[8], [9] These studies showed that human PDA also produces GM-CSF and one might envision a similar paucity of T cells in the tumor microenvironment. To evaluate the T cell and myeloid cell infiltrates in human PDA, we performed IHC for CD3 and CD11b in resected tumors from 40 patients (Table 1). We used inForm advanced image analysis software for all IHC quantifications, and found that the software precisely segmented both DAB and purple stained cells (Figure S1A–C).

**Table 1:** Patient and Tumor Characteristics.

Characteristic	No Therapy (N = 19)	Therapy (N = 21)
Age – yr		
Median	70	65
Range	45–82	40–78
Sex – no. (%)		
Female	6 (32)	10 (48)
Male	13 (68)	11 (52)
Preoperative biliary stent – no. (%)		
Yes	12 (63)	17 (81)
No	7 (37)	4 (19)
Level of carbohydrate antigen 19–9		
Mean U/mL	724	808
SD U/mL	1448	1280
Pretreatment T stage – no. (%)		
T1	2 (11)	0
T2	9 (47)	7 (33)
T3	8 (42)	14 (67)
Pretreatment N stage – no. (%)		
N0	16 (84)	11 (52)
N1	3 (16)	10 (48)
Pretreatment resectability – no. (%)		
Resectable	15 (79)	6 (29)
Borderline/Locally advanced	4 (21)	15 (71)
Surgical resection – no. (%)		
Pancreaticoduodenectomy	16 (84)	17 (81)
Distal pancreatectomy	2 (11)	3 (14)
Total pancreatectomy	1 (5)	1 (5)
Vascular resection – no. (%)		
Yes	4 (21)	5 (24)
No	15 (79)	16 (76)
Pathologic T stage – no. (%)		
T1	0	4 (19)
T2	1 (5)	8 (38)
T3	18 (95)	9 (43)
Nodes harvested – no.		
Median	15	11
Range	3–29	0–23
Pathologic N stage – no. (%)		
N0	3 (16)	16 (76)
N1	16 (84)	5 (24)
Surgical margin – no. (%)		
R0	11 (58)	16 (76)
R1	7 (37)	5 (24)
R2	1 (5)	0

**Figure 1:**
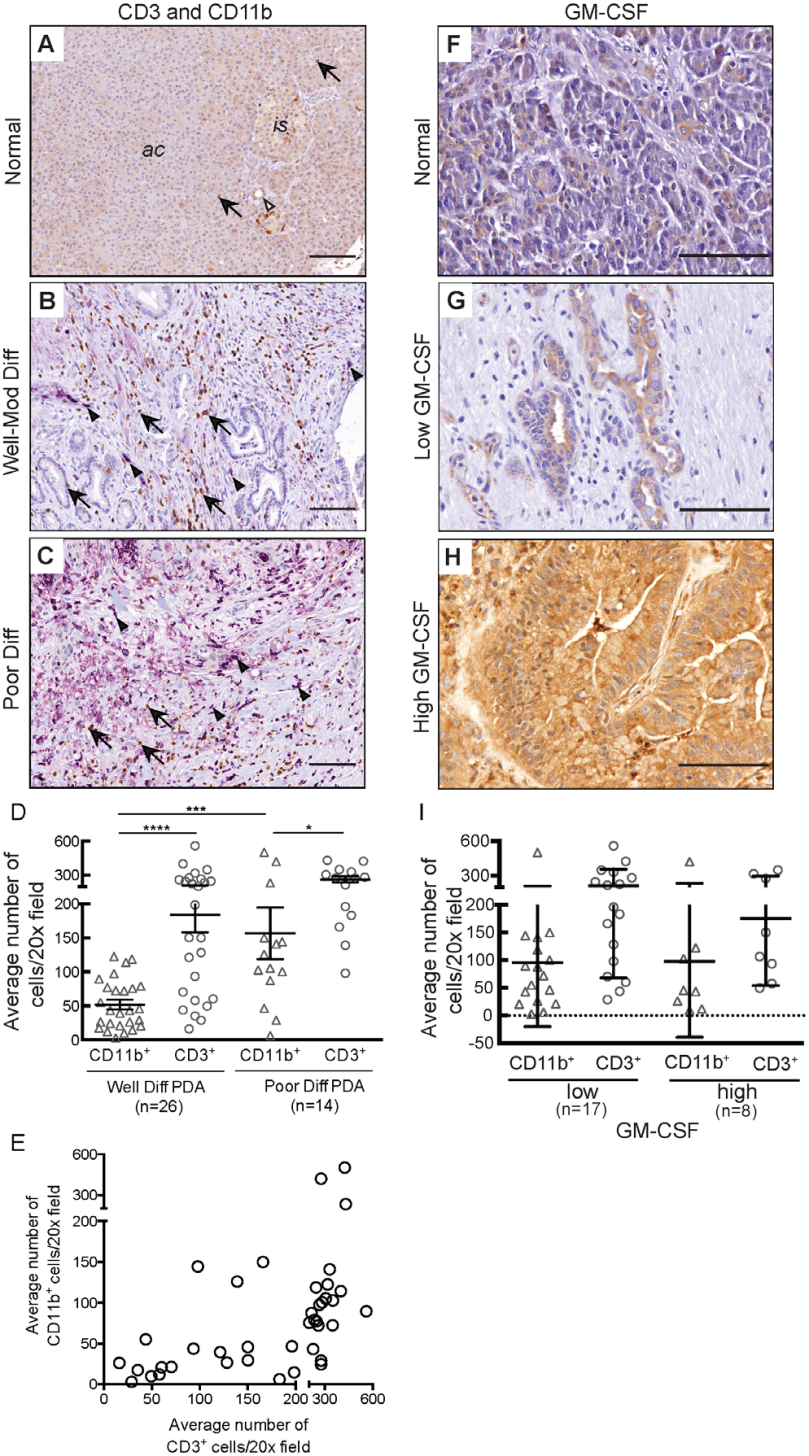
T cells infiltrate PDA regardless of GM-CSF expression. **A-C)** IHC of normal adjacent pancreas, well-differentiated PDA, and poorly differentiated PDA showing dual staining of T cells (CD3^+^ in brown, arrows) and myeloid cells (CD11b^+^in purple, arrowheads). Open arrowhead indicates a normal duct. Photomicrographs taken with a 20x objective. **D)** Quantification of CD3^+^ and CD11b^+^ cells in PDA (p = 0.0009). **E)** Correlation between density of CD3^+^ and CD11b^+^ cells in each tumor (r = 0.51; p = 0.0008). **F-H)** Representative photomicrographs (40x) showing GM-CSF expression in normal pancreas and PDA. **I)** Distribution of myeloid cells and T cells stratified based upon chromogen intensity for GM-CSF. At least five 40x fields were analyzed per specimen. Scale bars = 25 µm.

We found that, while T cells and myeloid cells were rare in normal, non-neoplastic pancreatic parenchyma (Figure 1A), both cell types were present both within the stroma and adjacent to carcinoma cells throughout PDA tumors (Figure 1B&C). Since tumor differentiation in PDA has been shown to impact survival,[26] we categorized tumors based upon standard histological criteria into well- to moderately (well-mod) differentiated or poorly differentiated. We determined the average numbers of CD3^+^ and CD11b^+^ cells in regions containing carcinoma cells and found that T cells were significantly more prevalent than myeloid cells in both well-mod differentiated (p<0.0001) and poorly differentiated tumors (p = 0.03; Figure 1D). There were significantly more myeloid cells in poorly differentiated tumors (p = 0.04), as well as a trend towards an increase in T cell infiltrate (p = 0.06) based upon tumor differentiation status. Importantly, we discovered a strong positive correlation between the density of T cell and myeloid cell infiltration in our specimens (r = 0.51; p = 0.0008; Figure 1E).

We stained a subset of the specimens for GM-CSF to determine if there was a relationship between its expression in human PDA and the character of the immune infiltrate. Normal pancreas tissue showed minimal GM-CSF expression (Figure 1F), while PDA tumors had a range of expression from low (Figure 1G) to high (Figure 1H). There was no association between intratumoral GM-CSF expression and the density of either the T cell or myeloid cell infiltrate (Figure 1I), nor was a correlation observed between the intensity of GM-CSF staining and the differentiation status of tumor (Figure S1D). Furthermore, we often observed CD3 positive cells overlying cancer glands in tumors with strong GM-CSF expression (Figure S2), demonstrating that tumor-derived GM-CSF production does not directly or indirectly preclude T cell homing and infiltration in human PDA.

### The T cell Infiltrate in PDA is Skewed towards CD8^+^ Effector Memory Cells and CD4^+^ Regulatory T cells

**Figure 2:**
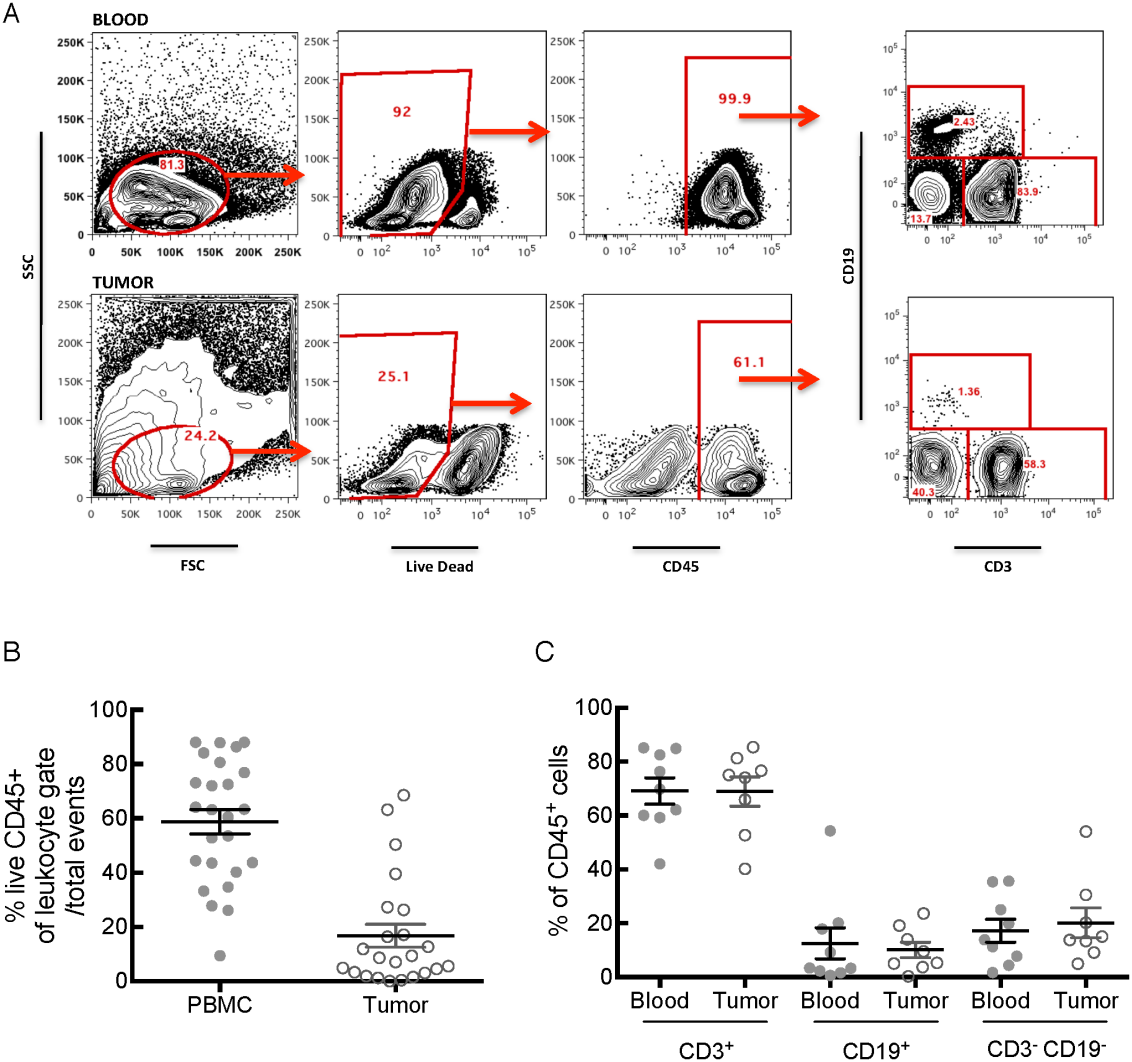
T cells dominate the intratumoral immune cell infiltrate. **A**) Representative flow cytometry plots illustrating the typical gating strategy used to identify the viable immune cells in blood and tumor. **B**) The proportion of live CD45^+^ cells was calculated for each specimen. **C**) CD3^+^ T cells consistently comprised the majority of the viable immune cells in blood and tumor, while CD19^+^ B cells and non-T or B cells comprise smaller proportions.

To confirm the results of IHC and characterize the phenotypic composition of the mononuclear and T cell infiltrates in relation to circulating cells in the blood, we performed flow cytometry on single-cell suspensions of digested tumor specimens and on matched peripheral blood samples procured at the time of surgical resection. Within the tumor specimens there was typically a large amount of debris, however after scatter gating and elimination of dead cells we defined a clear population of cells expressing the leukocyte common antigen CD45 (Figure 2A). Accurate analysis of the mononuclear cell subtypes within tumors is critically dependent on first eliminating dead cells and then staining with CD45, to distinguish hematopoietic derived cells from tumor cells that fall into the forward scatter/side scatter gate typically used to identify mononuclear cells (Figure 2B). Analysis of the CD45^+^ cells in the tumor and blood showed that the majority of these cells in both blood and tumor were CD3^+^, with smaller populations of CD19^+^ B cells and other non-T or B cells (Figure 2B&C). These numbers correlate well with our findings from IHC, thereby confirming that T cells are the predominant cell type infiltrating human PDA. Although we did not specifically identify myeloid cells within the single-cell suspensions of tumor cells, based upon our quantification from IHC these cells are likely to comprise a large proportion of the CD3^−^CD19^−^ cells.

**Figure 3:**
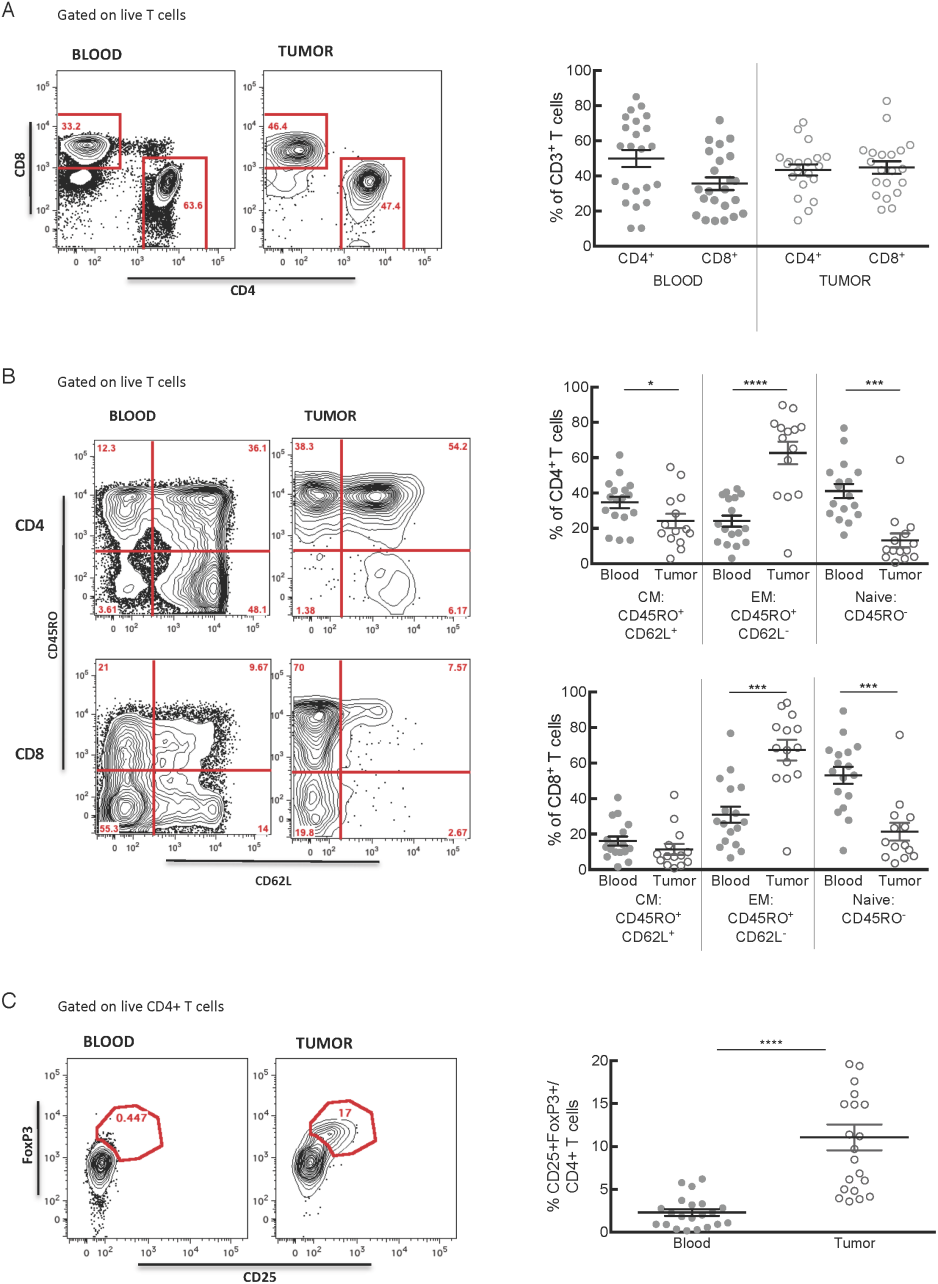
The majority of intratumoral T cells are antigen-experienced memory cells or regulatory cells. **A**) Both CD4^+^ and CD8^+^ T cells infiltrate PDA. **B**) Representative plots and summed data of central memory (CM), effector memory (EM), and naïve CD4^+^ and CD8^+^ T cell populations in the blood and tumor among live CD3^+^ T cells. Tumors contained significantly more CD4^+^ (p<0.0001) and CD8^+^ (p = 0.0001) EM cells than did the blood, while containing fewer CD4^+^ CM (p = 0.01), CD4^+^ naïve (p = 0.0003) and CD8^+^ naïve (p = 0.0007) T cells. **C**) After gating on live CD3^+^ CD4^+^ T cells, we used CD25 and FOXP3 to identify putative regulatory T cells (p<0.0001).

We next characterized the phenotype of the tumor-infiltrating CD3^+^ T cells and matched blood samples. Interestingly, the average CD4:CD8 ratio in the tumors was 0.97∶1, which was less than the ratio of 1.4∶1 in the blood, suggesting that CD8 T cells were relatively enriched in PDA tumors (Figure 3A). In the murine model data, the few T cells that infiltrate PDA tumors are typically of a naïve phenotype, and we hypothesized that this would be the case in human tumors as well. We found however that the majority of both CD4^+^ and CD8^+^ T cells infiltrating human PDA expressed the memory T cell marker CD45RO (Figure 3B), and most of these cells had an effector memory (EM, CD45RO^+^CD62L^−^) phenotype. The proportion of CD45RO^+^ CD62L^−^ cells was significantly greater in both CD4^+^ (p<0.0001) and CD8^+^ (p = 0.0001) subsets in the tumor compared with the blood (Figure 3B). Consistent with the elevated EM cell levels in tumor, naïve (CD45RO^−^) CD4^+^ (p = 0.0003) and CD8^+^ (p = 0.0007) T cells were significantly less prevalent in tumor than in the blood. A lower frequency of CD4^+^CD45RO^+^CD62L^+^ central memory T cells (CM) was also present in tumors than blood (p = 0.01), however there was no difference in CD8^+^ CM cells, which were infrequent in both compartments. Taken together, these data demonstrate that the T cell infiltrate in human PDA tumors is primarily composed of effector memory CD8^+^ and CD4^+^ T cells with a paucity of naïve T cells.

Prior reports have demonstrated infiltrates of Treg in both human and murine PDA based on immunohistochemical detection of FOXP3, the transcription factor that is critical for Treg development and function.[11], [13] Consistent with these studies, we routinely detected populations of CD4^+^ Treg (CD25^+^FOXP3^+^CD127^−^) in the single cell suspensions prepared from human PDA, and the proportion of Treg among CD4^+^ T cells in the tumor microenvironment was significantly higher than in the blood (p<0.0001; Figure 3C).

**Figure 4:**
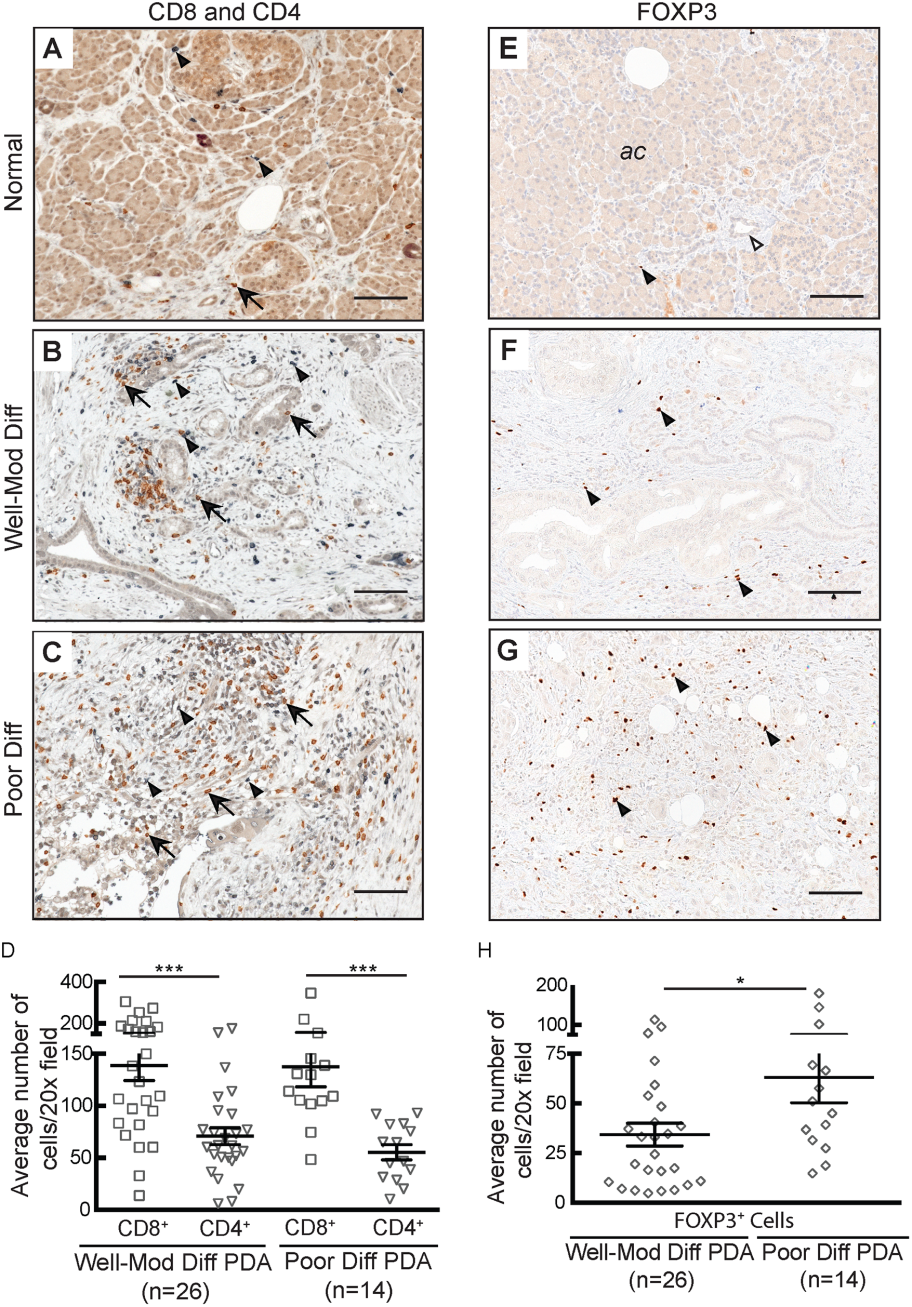
Histologic localization of cellular subtypes confirms the flow cytometric analysis of the immune infiltrate in PDA. **A–C**) Representative sections of normal pancreas and PDA showing multicolor IHC for CD8^+^ (brown, arrows) and CD4^+^ (purple, arrowheads) cells. **D**) Quantification of CD8^+^ (p = 0.0001) and CD4^+^ (p = 0.0005) cells. **E–G**) Representative sections of normal pancreas and PDA stained for FOXP3. Arrowheads indicate FOXP3^+^ cells, open arrowhead demonstrates a normal duct. **H**) Quantification of FOXP3^+^ cells (p = 0.02). At least five 20x fields were analyzed per specimen per cell type. Scale bars = 25 µm.

We performed multi-color IHC on serial sections of our paraffin-embedded tumors to determine if the findings on T cell subset distribution from tumor digests could be confirmed. Consistent with the flow cytometry and IHC for CD3, both CD4^+^ and CD8^+^ T cell subsets were rare in normal pancreatic parenchyma (Figure 4A) and were plentiful in pancreatic cancer (Figure 4B&C). We detected significantly more CD8^+^ than CD4^+^ T cells in both well-differentiated (p<0.0001) and poorly differentiated (p = 0.001) tumors (Figure 4D), confirming our prior finding that CD8^+^ T cells are enriched in the tumor microenvironment relative to the blood. FOXP3 staining was extremely rare in normal pancreas (Figure 4E), but frequently co-localized to regions containing CD4^+^ T cells in PDA tumors (Figure 4F&G). While we were unable to detect a difference in the density of either CD4^+^ or CD8^+^ cells in PDA tumors based upon differentiation status, there were significantly more FOXP3^+^ cells in poorly differentiated tumors (p = 0.03; Figure 4H). The consistent presence and close anatomic proximity of the varied cellular subtypes further support the idea that a complex T cell immune response containing both effector and regulatory components is a defining feature of human PDA.

### T cells in the Tumor Microenvironment Retain the Capacity to Produce Effector Cytokines, but Express the Co-inhibitory Receptor PD-1

**Figure 5:**
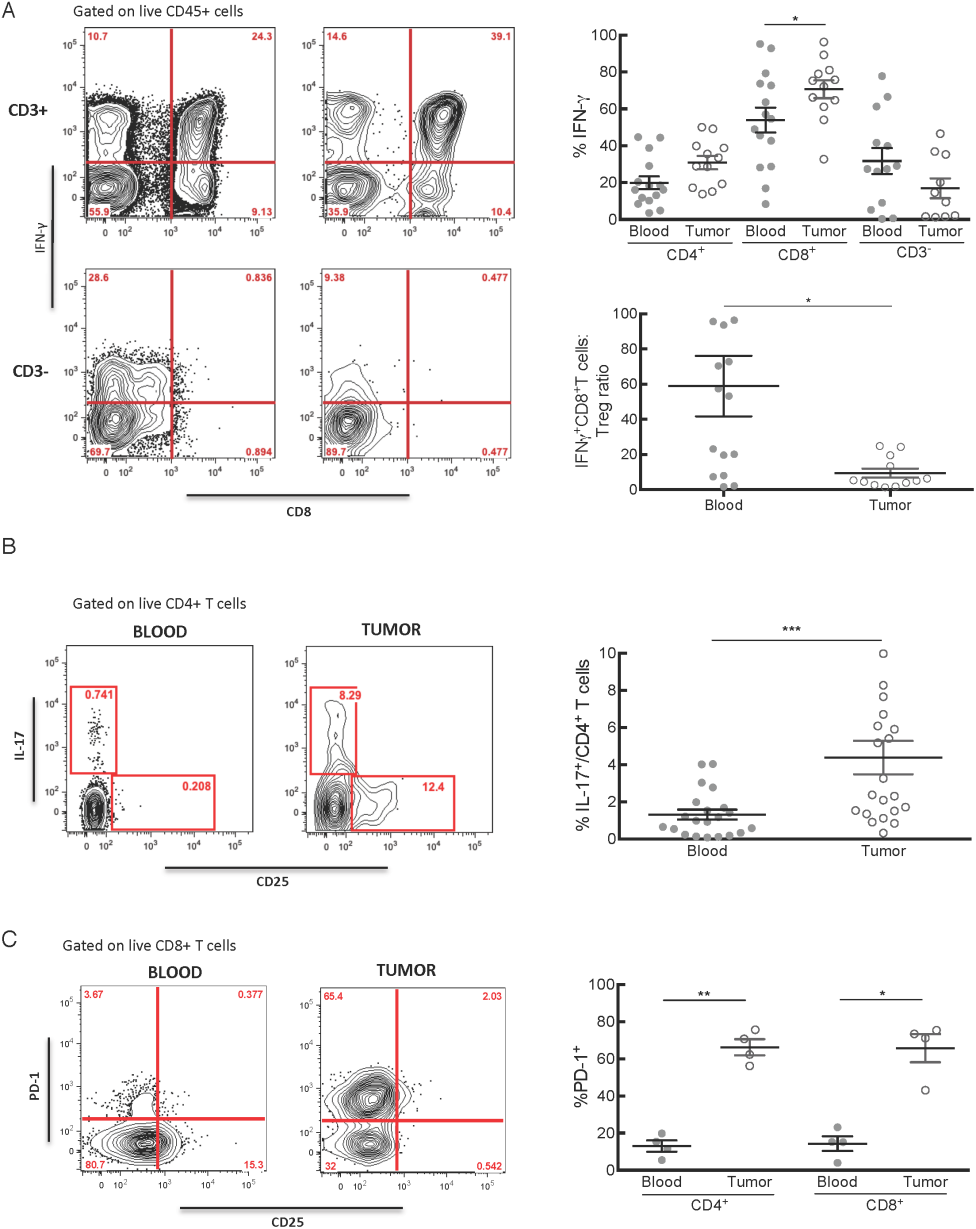
T cells in the tumor microenvironment retain functional capacity to activate inflammation despite the presence of multiple potential regulatory mechanisms. A) Single-cell tumor suspensions and PBMC isolated from matched blood samples were incubated with PMA/ionomycin for 4 hours prior to staining for multiparameter flow cytometry. Staining for intracellular IFN-γ was positive in a significantly higher proportion of CD8^+^ T cells in tumor than in blood (p = 0.03), and there was a similar trend among CD4^+^ T cells (p = 0.05). A small proportion of CD3^−^ cells thought to represent NK cells produced IFN-γ. The ratio of IFN-γ producing CD8^+^ T cells to Treg in blood and tumor is shown (p = 0.02). B) Th17 cells were identified by gating on live IL-17^+^CD4^+^ T cells. (p = 0.0008). C) Plot showed expression of PD-1 by both CD4^+^ and CD8^+^ unstimulated T cells (p = 0.003 and 0.02, respectively).

The large proportion of effector memory T cells in the tumor microenvironment raised the question as to whether these T cells were capable of effector functions, or might be functionally exhausted due to local regulatory and immune suppressive mechanisms, or to chronic antigen stimulation as observed in some persistent viral infections.[27] Since antigen specificity of the infiltrating T cells is unknown, we used non-specific stimulation with PMA/ionomycin to determine if these T cells retained the ability to mediate effector functions. We found that the majority of CD8^+^ T cells and a significant fraction of CD4^+^ T cells infiltrating the tumors were capable of producing IFN-γ (Figure 5A). Furthermore, there was a significantly higher frequency of IFN-γ producing CD8^+^ T cells in tumors than in blood (p = 0.03), and a similar trend was observed in the CD4 T cell compartment (p = 0.05).

We determined the ratio of effector to regulatory T cells since this may be decisive in determining whether an anti-tumor immune response is effective.[28] The mean ratio of IFN-γ^+^ CD8^+^ T cells to Treg in blood was 60.0, SD 66.6 and in tumors was 9.4, SD 8.8 (p = 0.02) (Figure 5A). These data suggest that despite a large infiltrate of T cells that are capable of effector function in PDA, there remains a bias towards immune suppression in the tumor microenvironment, due in part to a relative increase in the numbers of CD4^+^ Treg.

Th17 cell have been implicated in a variety of inflammatory conditions and are postulated to have a role in many epithelial cancers including PDA.[29]–[31] To determine if a subset of the FOXP3^−^CD4^+^ T cells might produce IL-17, we performed intracellular cytokine staining for IL-17 after PMA/ionomycin stimulation. We identified a small fraction of IL-17 producing CD4^+^ T cells in the tumor infiltrate and these comprised a significantly higher proportion of CD4^+^ T cells in tumor than in blood (p = 0.0008; Figure 5B).

A variety of receptor to ligand interactions also regulate T cell immunity, and many of these are co-opted by tumor cells to suppress local T cell responses. The PD-1:PD-L1 pathway has been identified as one of the important pathways for impeding effective immune responses to several human cancers, including non small cell lung cancer that was previously not thought to be a likely candidate for immunotherapy.[23], [24] It has previously been demonstrated that human PDA expresses PD-L1,[32] however the level of expression of PD-1 on T cells in PDA is not known. We therefore measured PD-1 expression by CD8^+^ and CD4^+^ T cells in the blood and tumors of patients with PDA and determined that there was a markedly higher proportion of PD-1^+^ CD4^+^ and CD8^+^ T cells in tumors compared to paired blood samples (p = 0.003 and 0.02, respectively; Figure 5C). These results suggest that multiple regulatory mechanisms are operative in suppressing the local function of infiltrating T cells in PDA.

### Successful Multimodal Neoadjuvant Therapy Is Associated with a Reduced Number of Potentially Immunosuppressive Cell Types in the Tumor Microenvironment

**Figure 6:**
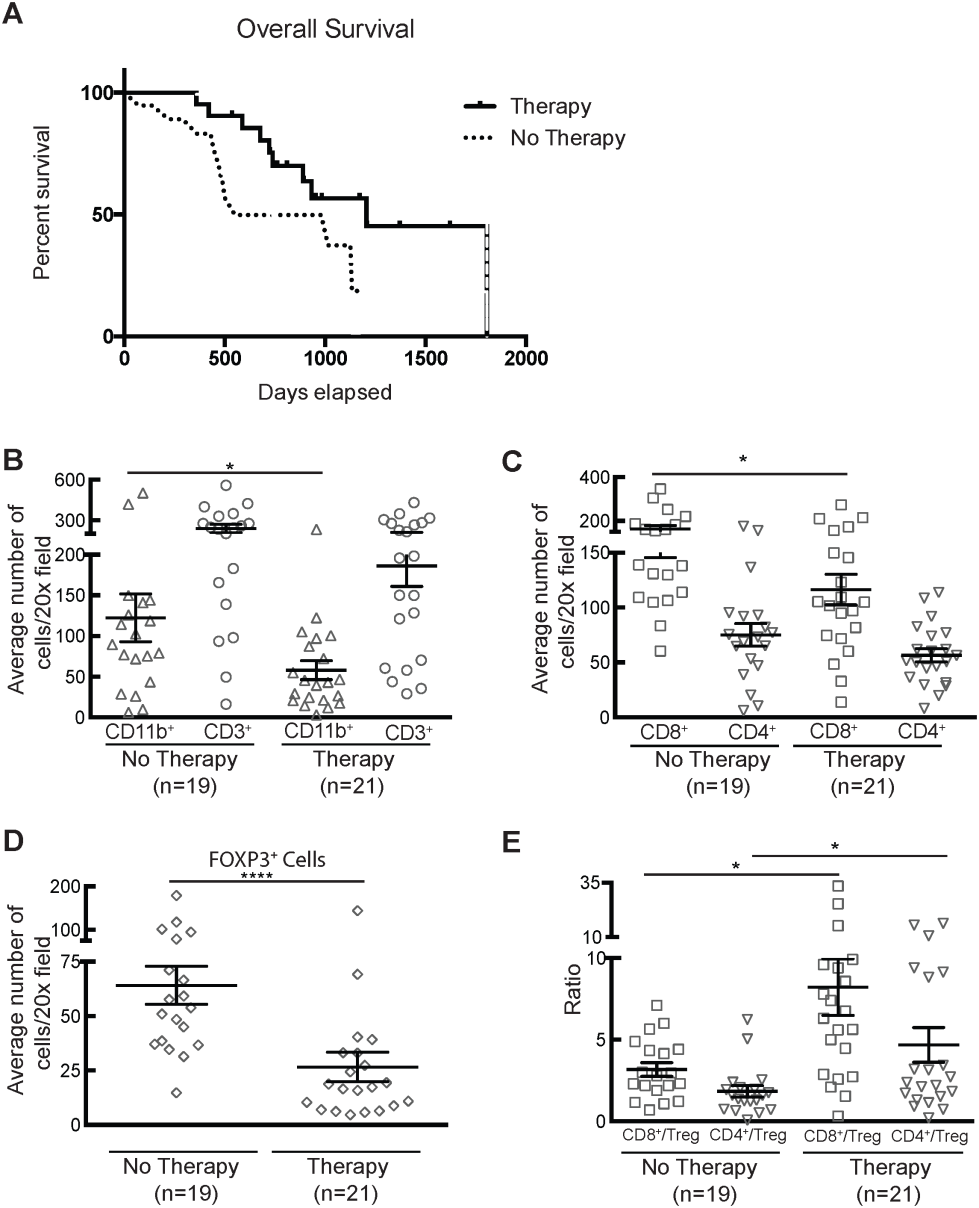
Multimodal neoadjuvant therapy alters the regulatory cell makeup of the tumor microenvironment. **A**) Kaplan-Meier survival of patients in this study. There was a trend towards longer survival among patients undergoing neoadjuvant therapy (p = 0.07). **B**) Quantification of CD11b^+^ (p = 0.04) and CD3^+^cells in neoadjuvant therapy vs. untreated tumors. **C**) CD4^+^ cells were unchanged, however CD8^+^ cells were less frequent after neoadjuvant therapy (p = 0.04). **D**) Quantification of FOXP3^+^ cells in treated and untreated tumors (p = 0.002). **E**) Analysis of ratio of CD8^+^ to FOXP3^+^ T cells (p = 0.01) and CD4^+^ to FOXP3^+^ T cells (p = 0.01) in treated and untreated tumors.

As shown in Table 1, approximately half of the 40 IHC specimens in this study are from patients who received neoadjuvant therapy consisting of systemic gemcitabine-based chemotherapy plus chemoradiotherapy (n = 19), or chemotherapy alone (n = 2). Neoadjuvant therapy has previously been shown to alter immune populations in peripheral blood of patients with PDA, leading to transient changes in DC subtypes, total T cell population as well as memory cells[33] and a significant reduction in Treg.[34] Since the neoadjuvant treated patients in our study had a trend towards better overall survival than did patients who underwent primary surgical resection (p = 0.07; Figure 6A), we hypothesized that there would be associated differences in the immune infiltrate. We found significantly fewer myeloid cells infiltrating tumors from patients who had received neoadjuvant therapy compared to those who underwent primary surgery (p = 0.04), however there was no difference in numbers of infiltrating CD3^+^ T cells (Figure 6B). Treated tumors had a somewhat lower average number of CD8^+^ cells than did untreated ones (p = 0.04), while there was no difference in the number of CD4^+^ cells (Figure 6C). Importantly, there was significantly less infiltration of treated tumors with FOXP3^+^ cells (p = 0.002; Figure 6D). Accordingly, the CD8:Treg and CD4:Treg ratios were higher in treated tumors (p = 0.01; Figure 6E), suggesting that preoperative therapy may specifically deplete immunosuppressive cell types in the PDA tumor microenvironment.

## Discussion

The immune system is known to be intimately involved in the development, progression or control of multiple cancers, including PDA.[35] Prior studies identified T cells infiltrating human PDA and demonstrated a positive relationship between the density of the T cell infiltrate and survival.[2], [11] In the present study, we used IHC and flow cytometry to characterize the immune infiltrate in PDA from both a qualitative and quantitative standpoint. Our study differs from prior work in several respects. Firstly, we used quantitative methods including flow cytometry and automated cell counting of IHC stained specimens to evaluate the immune infiltrate. Secondly, we characterized in detail the phenotype and function of tumor infiltrating T cells in comparison to T cells from matched peripheral blood. Lastly, our study included nearly equal numbers of tumors from patients who did or did not receive multimodal neoadjuvant therapy, which permitted an analysis of how immune infiltrates may be altered by chemotherapy and chemoradiotherapy. We found that, although myeloid cells increasingly infiltrate poorly differentiated tumors, T cells are consistently the predominant CD45^+^ hematopoietic cell type in PDA tumors regardless of the differentiation status of the tumor. The tumor-infiltrating T cells included a robust population of effector memory phenotype CD8^+^ T cells, and large numbers of CD4^+^ Treg. Importantly, multimodal neoadjuvant therapy altered the regulatory balance through selective depletion of potentially suppressive myeloid cells and Treg.

Our findings contrast with the prevailing view of the immune response to PDA that is based largely upon studies in GEMM in which T cells rarely infiltrate murine tumors and mostly remain sequestered in the peripancreatic lymph nodes.[7] Although MDSC recruited by tumor-derived GM-CSF have been shown to inhibit T cells from entering murine PDA tumors,[8], [9] our analysis of human PDA demonstrates that, while these tumors also produce GM-CSF, they contain both T cells and myeloid cells tightly juxtaposed in the tumor microenvironment. Moreover, the numbers of T cells and myeloid cells correlate closely. In accordance with their antigen-experienced phenotype, we found that the majority of the CD8^+^ T cells and a portion of the CD4^+^ T cells were capable of producing IFN-γ. These differences between the immune response to pancreatic cancer in the model system and patients suggests that caution be applied in extrapolating findings between systems, particularly when it comes to planning clinical trials of immunotherapy. The discrepancy may be related to the heightened biologic complexity and cellular heterogeneity that results from the lengthy evolution of clinical pancreatic cancer, compared to the murine model.

Despite the presence of an effector T cell infiltrate in human PDA, survival is dismal, and we hypothesized that multiple regulatory mechanisms might account for the apparent lack of an effective immune response. In accordance with prior murine and human studies,[6], [11] we confirmed the presence of FOXP3^+^ Treg in PDA tumors and made the new observation that they are markedly enriched in the tumor microenvironment relative to the blood. Concomitant with our finding of elevated Treg infiltrates in more poorly differentiated tumors, we noted a similar relationship between differentiation status and myeloid cell infiltrate. These data fit well with a recent publication that has demonstrated a positive impact of CD4+ and CD8+ T cell infiltration on survival in PDA, as well as a negative impact of both Treg and specific myeloid cell infiltrates.[2] Due to our smaller total number of patients and heterogeneity in treatment strategy, we are unable to demonstrate a similar independent prognostic value of the immune infiltrate.

The immune checkpoint ligand PD-L1 has been shown to be expressed in PDA[36] and by tumor-associated macrophages[37], suggesting infiltrating T cells might be subject to regulation through PD-1. Indeed, we found that the majority of both CD4^+^ and CD8^+^ T cells in the tumor microenvironment of PDA expressed PD-1, in contrast to the rarity of PD-1 expressing T cells in peripheral blood. Our observation that the majority of intratumoral CD8^+^ T cells are capable of IFN-γ production suggests that regulation by PD-L1/PD-1 interactions might inhibit local effector function. If this were the case, one might reasonably anticipate that blockade of the PD-1 pathway would provide clinical benefit. However, PD-1 pathway blockade has so far been unsuccessful in the treatment of a small number of patients with advanced PDA.[24] One plausible explanation for this lack of response in PDA patients is that the tumor infiltrating T cells may require blockade of additional immune checkpoints such as T cell immunoglobulin mucin 3 (Tim-3), LAG-3 or CTLA4 to restore anti-tumor function.[38] Alternatively, liver metastases – the most common cause of mortality in PDA – may be comprised of an entirely different immune microenvironment influenced by the uniquely tolerogenic milieu of the liver. In future work, we plan to delineate the expression of other co-inhibitory receptors on T cells in primary tumors and hepatic metastases from patients with PDA to inform the rational use of combinations of checkpoint blockade antibodies, which is an approach that has recently demonstrated efficacy in the treatment of melanoma.[39].

Although surgical resection remains the standard initial treatment of early-stage PDA, neoadjuvant therapy has some theoretical and practical advantages.[40], [41] Previous studies have reported immunomodulatory effects of chemotherapy reagents including enhancement of CD8^+^ T cell function[42] and direct depletion of MDSC and Treg.[43] A recent study demonstrated reduction in the numbers and percentage of Treg in peripheral blood of patients undergoing gemcitabine-based chemotherapy for PDA.[34] In the present study, we made the new observation that multimodal neoadjuvant treatment of PDA results in a significantly lower number of myeloid cells and Treg in the tumor microenvironment. Despite a significant reduction in CD8^+^ cells with therapy, the ratios of both CD4^+^ and CD8^+^ cells to Treg were significantly higher in treated tumors. The relative depletion of potentially immunosuppressive cells in the tumor microenvironment of PDA makes combination of standard neoadjuvant therapies with such immunotherapeutic strategies as immune checkpoint blockade or adoptive cellular therapy, as we are pursuing in our laboratory, particularly appealing.

Since the two patient groups were not prospectively matched, one concern related to these data might be that our findings resulted from selection bias derived from the decision of whether or not to perform neoadjuvant therapy. Importantly, many of the patient characteristics, including age, sex, preoperative biliary stent placement, type of surgical procedure performed, and initial level of serum carbohydrate antigen 19–9, were similar. However, there were notable differences in that patients treated with neoadjuvant therapy initially had more advanced disease characterized by higher rates of clinical positive lymph nodes (p = 0.03) and were more likely to have borderline resectable or locally advanced disease (0.0009). Speaking to the efficacy of the neoadjuvant therapy, final pathologic evaluation showed the opposite relationship, as treated tumors were less frequently T3 (p = 0.0002) or lymph node positive (p<0.0001).

Collectively, our data show that the tumor microenvironment of human PDA is characterized by a dense T cell predominant immune infiltrate that includes both effector and regulatory cell subsets. The presence of a large proportion of CD8^+^ effector memory T cells capable of IFN-γ production suggests that there may be an active immune response in the tumor microenvironment that is suppressed by CD11b^+^ and FOXP3^+^ cells and by interactions with cells that express ligands such as PD-L1 for inhibitory receptors on T cells. Importantly, we show that neoadjuvant treatment of PDA with chemotherapy and chemoradiotherapy can alter the regulatory balance in favor of antitumor immunity, and might be useful in combination with immune checkpoint inhibitors. The poor prognosis of PDA with current therapy argues for additional investigation of the potential application of immunotherapy in this disease.

## Supporting Information

Figure S1 Representative photomicrographs showing multispectral image analysis using Nuance EX Multispectral Tissue Imaging Systems. A) Composite RGB image showing dual staining of T cells (CD3^+^ in brown, arrows) and myeloid cells (CD11b^+^ in purple, arrowheads) in normal human pancreas and PDA. B) Segmentation map of CD3^+^ stained with DAB. All DAB stained areas were filled with green color. **C**) Segmentation map of CD11b^+^ stained with purple. All purple stained areas were filled with green color. D) Quantification of the chromgen intensity of GM-CSF in well- and poorly differentiated tumors (p = 0.7). Scale bars = 25 µm.
(TIFF)

Figure S2 Representative photomicrographs (serial sections) from two different PDA cases showing CD3^+^ cells (arrows) cells in close proximity to cells expressing GM-CSF (open arrowheads). Scale bars = 25 µm.
(TIFF)
