# Supplementary figures and images for: Pancreatic Ductal Adenocarcinoma Contains an Effector and Regulatory Immune Cell Infiltrate that Is Altered by Multimodal Neoadjuvant Treatment
Source: PLoS One. 2014 May 2;9(5):e96565. doi: 10.1371/journal.pone.0096565 (PMC4008589; doi:10.1371/journal.pone.0096565)

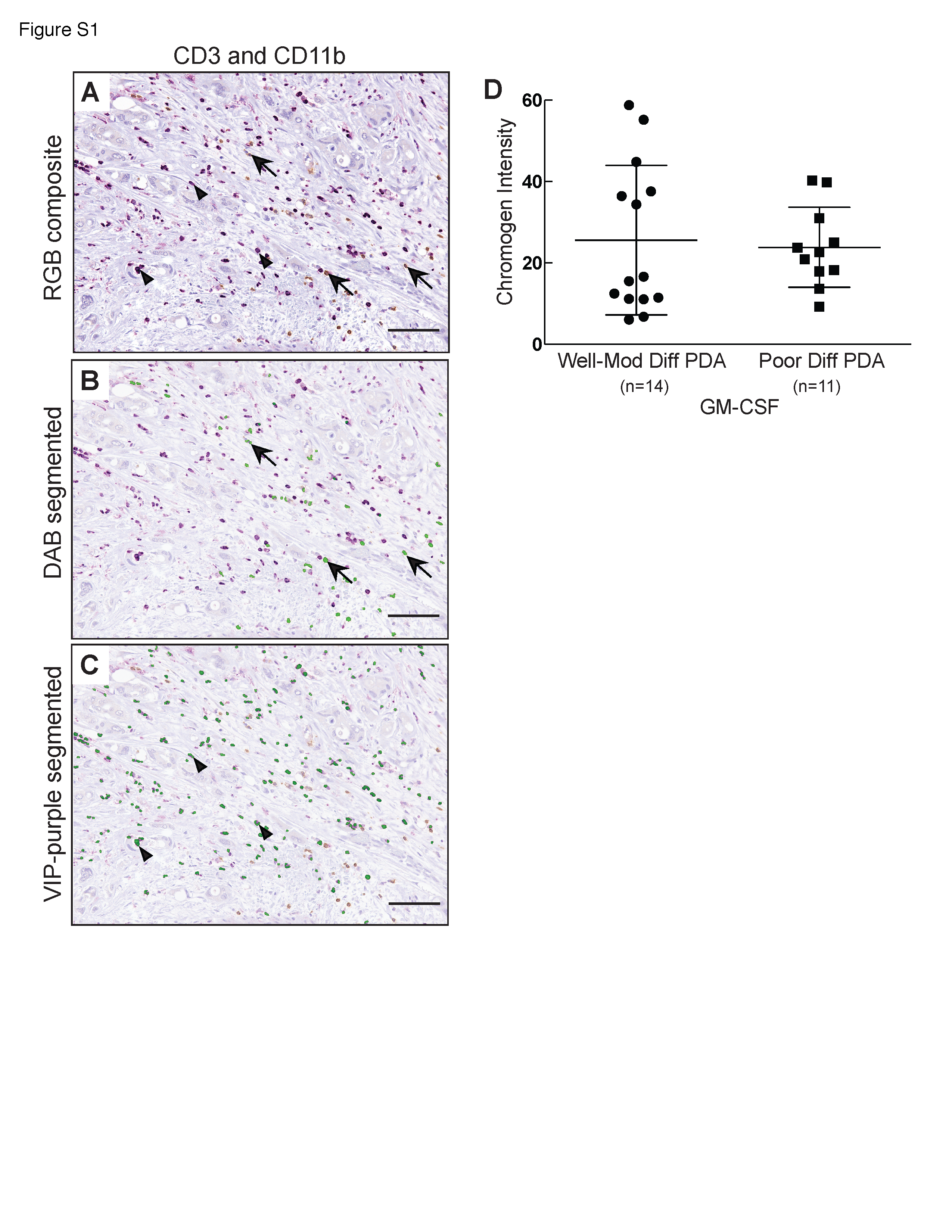

Supplement: Figure S1 — Representative photomicrographs showing multispectral image analysis using Nuance EX Multispectral Tissue Imaging Systems. A) Composite RGB image showing dual staining of T cells (CD3+ in brown, arrows) and myeloid cells (CD11b+ in purple, arrowheads) in normal human pancreas and PDA. B) Segmentation map of CD3+ stained with DAB. All DAB stained areas were filled with green color. C) Segmentation map of CD11b+ stained with purple. All purple stained areas were filled with green color. D) Quantification of the chromgen intensity of GM-CSF in well- and poorly differentiated tumors (p = 0.7). Scale bars = 25 µm. (TIFF) [file pone.0096565.s001.tiff]

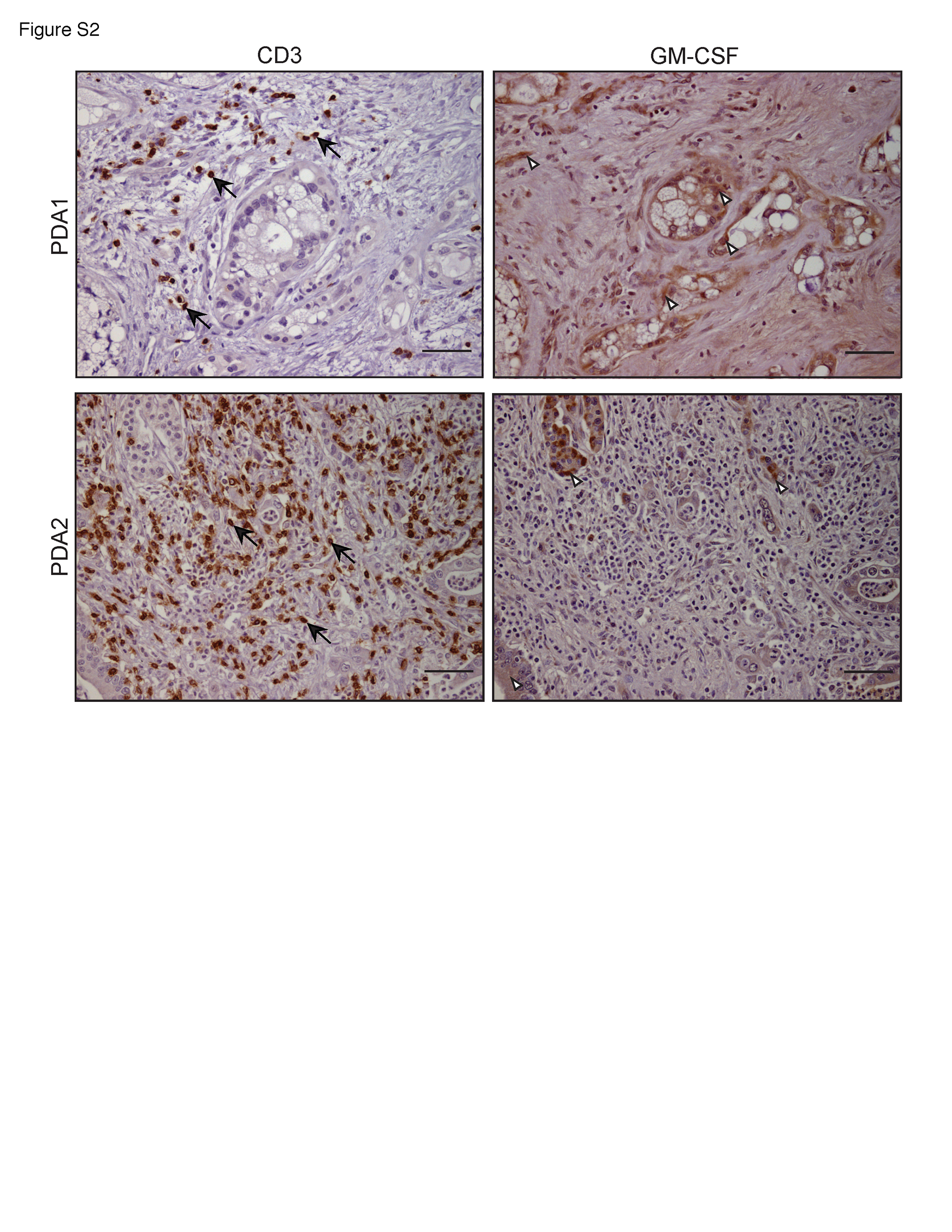

Supplement: Figure S2 — Representative photomicrographs (serial sections) from two different PDA cases showing CD3+ cells (arrows) cells in close proximity to cells expressing GM-CSF (open arrowheads). Scale bars = 25 µm. (TIFF) [file pone.0096565.s002.tiff]
